# A Mutant *Ahr* Allele Protects the Embryonic Kidney from Hydrocarbon-Induced Deficits in Fetal Programming

**DOI:** 10.1289/ehp.1103692

**Published:** 2011-07-29

**Authors:** Adrian Nanez, Irma N. Ramos, Kenneth S. Ramos

**Affiliations:** 1 Department of Biochemistry and Molecular Biology, and; 2 Department of Environmental and Occupational Health Sciences, University of Louisville, Louisville, Kentucky, USA

**Keywords:** aryl hydrocarbon receptor, benzo(*a*)pyrene, fetal programming, nephrogenesis, WT1

## Abstract

Background: The use of experimental model systems has expedited the elucidation of pathogenetic mechanisms of renal developmental disease in humans and the identification of genes that orchestrate developmental programming during nephrogenesis.

Objectives: We conducted studies to evaluate the role of *AHR* polymorphisms in the disruption of renal developmental programming by benzo(*a*)pyrene (BaP).

Methods: We used metanephric cultures of C57BL/6J (C57) mice expressing the *Ahr^b-1^* allele and B6.D2N-*Ahr^d^*/J (D2N) mice expressing a mutant allele deficient in ligand binding (*Ahr^d^*) to investigate molecular mechanisms of renal development. Deficits in fetal programming were evaluated in the offspring of pregnant mice treated with BaP during nephrogenesis.

Results: Hydrocarbon challenge of metanephri from C57 mice altered Wilms’ tumor suppressor gene (*Wt1*) mRNA splice variant ratios and reduced mRNAs of the *Wt1* transcriptional targets syndecan-1 (*Sdc1*) paired box gene 2 (*Pax2*), epidermal growth factor receptor (*Egfr*), and retinoic acid receptor, alpha (*Rar*α). These changes correlated with down-regulation of effectors of differentiation [secreted frizzled-related sequence protein 1 (*Sfrp1*), insulin-like growth factor 1 receptor (*Igf1r*), wingless-related MMTV-integration site 4 (*Wnt4*), Lim homeobox protein 1 (*Lhx1*), E-cadherin]. In contrast, metanephri from D2N mice were spared hydrocarbon-induced changes in *Wt1* splice variant ratios and deficits of differentiation. We observed similar patterns of dysmorphogenesis and progressive loss of renal function at postnatal weeks 7 and 52 in the offspring of pregnant C57 but not D2N mice gavaged with 0.1 or 0.5 mg/kg BaP on gestation days 10–13.

Conclusions: These findings support a functional link between AHR and WT1 in the regulation of renal morphogenesis and raise important questions about the contribution of human AHR polymorphisms to the fetal origins of adult-onset kidney disease.

Embryonic development requires the orchestration of temporally precise genetic events that culminate in formation of a complete organism and that respond to diverse environmental and somatic signals *in utero*. Previous studies identified the aryl hydrocarbon receptor (AHR) as an important nuclear transcription factor during mammalian embryogenesis and throughout maturity. Ligand-activated AHR interacts with hypoxia-inducible factor 1β (HIF1β) to form transcriptional complexes that bind specific DNA sequences to regulate genetic targets (Denison and Nagy 2003). Nuclear translocation triggers proteolytic degradation of AHR protein, a signaling event that defines the biological responsiveness to AHR ligands (Roberts and Whitelaw 1999). AHR protein is highly polymorphic in the core ligand-binding and transactivation domains, and this variability dictates differences in susceptibility to environmental injury in mice (Moriguchi et al. 2003) and humans (Sasaki et al. 2006).

AHR participates in developmental regulation of vascular structures in liver (Fernandez-Salguero et al. 1995; Lahvis et al. 2005; Schmidt et al. 1996), as well as morphogenesis of heart (Lund et al. 2003, 2006) and kidney (Falahatpisheh and Ramos 2003; McMillan and Bradfield 2007). A role for AHR in renal development was firmly established in experiments showing that *Ahr*-null mice exhibit deficits in renal condensation, appearance of differentiated structures, and cellular proliferation (Falahatpisheh and Ramos 2003). The kidney derives from the nephric ridge of the intermediate mesoderm where the pronephric duct elongates to form the Wolffian duct, which in turn gives rise to the ureteric bud and sets the stage for condensation of metanephric mesenchyme and mesynchemal-to-epithelial cell transition. Wilms’ tumor suppressor gene (*Wt1*) functions as a critical regulator of nephrogenesis by encoding a Cis_2_-His_2_ zinc-finger protein that functions via transcriptional regulation of insulin-like growth factor 2 (*Igf2r*), syndecan-1 (*Sdc1*), epidermal growth factor receptor (*Egfr*), and retinoic acid receptor, alpha (*Rar*α), among other targets (Avner 1993; Hosono et al. 1999). Homozygous *Wt1*^–/–^ mice do not undergo differentiation from pronephros to metanephros and die *in utero* (Kreidberg et al. 1993). *Wt1* activity can be regulated in *cis* by the different ratios of its own splice variants and in *trans* by proteins such as bone marrow zinc finger 2 (BMZF2) (Lee et al. 2002). The most studied regulatory mechanism of *Wt1* involves the formation of +KTS and –KTS splice variants. KTS splice variants originate from the insertion of a lysine-threonine-serine between the third and fourth zinc fingers, and this change regulates *Wt1* DNA binding specificity (Menke et al. 1998). In humans, reduced *WT1* +KTS mRNA isoforms result in severe kidney and gonad developmental deficits, known as Frasier syndrome (Barbaux et al. 1997). Changes in exon 5 splice variants are also associated with deficits in renal differentiation (Iben and Royer-Pokora 1999). Addition of 17 amino acids in exon 5 creates an mRNA isoform (17aa) that regulates transactivation (Wang et al. 1995). N-terminal residues 1–182 encode a dimerization region implicated in the regulatory mechanism exerted by dominant negative mutants (Englert et al. 1995).

The precise mechanism by which AHR regulates genetic elements during nephrogenesis is not known. Given the requisite activation of AHR by endogenous or exogenous ligands, it is likely that developmental regulatory functions of AHR involve transcriptional regulation of genes during early kidney morphogenesis. The polymorphic nature of the AHR locus suggests that the inherent susceptibility of mice and humans to developmental interference by AHR ligands is variable. Such relationships can be studied taking advantage of murine models expressing variant AHR proteins that structurally and functionally resemble those in humans. The C57BL/6J (C57) mouse is perhaps the most widely used mouse model to evaluate the biology of AHR (Moriguchi et al. 2003). This strain is naturally sensitive to exogenous AHR ligands, as evidenced by transregulation of AHR-regulated genes (Moriguchi et al. 2003). In contrast, B6.D2N-*Ahr^d^*/J (D2N) mice are highly resistant to ligand-mediated AHR activation and gene transactivation. The C57 and D2N mice are isogenic strains that differ in AHR ligand-binding affinity due to a single nucleotide substitution that replaces valine for alanine at codon 375 in the ligand-binding domain, resulting in a 10-fold reduction in ligand-binding affinity (Poland et al. 1994). Humans predominantly express an AHR protein of reduced ligand-binding affinity similar to that in D2N mice, with several polymorphic variants identified to date that exhibit increased ligand-binding affinities and transactivation potentials (Sasaki et al. 2006). Some of these variants afford heightened susceptibility to cancer, and possibly developmental interference. We conducted the present study to evaluate the impact of polymorphic variants of the AHR on hydrocarbon-induced deficits in nephrogenesis. We present evidence that a mutant *Ahr* allele deficient in ligand-binding affinity and nuclear transactivation protects the developing murine kidney from hydrocarbon-induced deficits of fetal genetic programming and loss of renal function in adult life. These findings implicate AHR in the regulation of renal developmental programming and the fetal basis of adult-onset kidney disease.

## Materials and Methods

*Metanephric cultures.* On gestation day (GD) 11.5, mouse embryos were dissected from C57BL/6J *Ahr^b-1/b-1^* wild-type and B6.D2N-*Ahr^d/d^*/J mice (Jackson Laboratories, Bar Harbor, ME). All animals were treated humanely and with regard for alleviation of suffering. Metanephri were cultured on 0.45-mm polyethylene terephthalate cyclopore cell culture inserts (Fisher Scientific, Pittsburgh, PA) for 1–4 days. Kidney explants were maintained at the liquid–gas interface in a solution consisting of a 1:1 mixture of Dulbecco’s modified Eagle’s medium and F12 medium supplemented with 10% fetal bovine serum and a 5× concentration of MITO serum extender (Becton Dickenson, Bedford, MA). Explants were equilibrated for 1 day before start of experiments. Seven or more kidney explants from four dams were placed on individual inserts and exposed daily to 3 μM benzo(*a*)pyrene (BaP) or an equivalent volume of dimethyl sulfoxide (DMSO), with or without 20 nM α-napthoflavone (α-NF), for 1, 3, or 4 days. α-NF is a ligand of AHR that fails to induce conformational changes for efficient transactivation of target genes and thus functions as a competitive antagonist. The dose of BaP tested *in vitro* represents an environmentally relevant dose that affords optimal activation of AHR (Bowes and Ramos 1994). Explants were fixed *in situ* and processed for further evaluation.

*Intrauterine exposures to BaP.* Timed-pregnant C57BL/6J *Ahr^b-1/b-1^* and B6.D2N-*Ahr^d/d^*/J mice were gavaged on GDs 10–13 with 0.1 or 0.5 mg/kg BaP or medium-chain triglyceride oil (MCT; Mead Johnson Nutritionals, Evansville, IN). MCT is a well-characterized emulsion vehicle optimal for oral BaP formulations. The doses examined are considerably lower than those used previously in studies of teratogenicity (Lummus and Henningsen 1995; MacKenzie and Angevine 1981; Rodriquez et al. 1999; Wells et al. 1997; Winn and Wells 1997) and approximate human exposures among at-risk populations when corrected for differences in ontogenic profiles (Menzie et al. 1992; Rebagliato et al. 1995). One week after birth, some pups were euthanized and kidney, heart, liver, testis, and aorta were fixed *in situ* under physiological pressure; others were kept for studies of renal structure and function 52 weeks after birth.

*Histology and morphometric analysis.* Metanephri were fixed in 4% paraformaldehyde at 4°C for 16 hr, immobilized in Histogel (Richard Allan Scientific, Kalamazoo, MI), and embedded in paraffin. Serial sections (4 μm) were cut and stained with hematoxylin and eosin (H&E) for visualization of differentiated structures. Images of at least five metanephri per treatment group were captured with an Axiovert 200 inverted microscope (Carl Zeiss Microscopy, Thornwood, NY) and stored as ZVI files. Glomeruli and S-shaped and comma-shaped bodies were quantified using manual functions in AxioVision (release 4.1; Carl Zeiss Microscopy). For *in vivo* measurements, kidneys were fixed in 4% paraformaldehyde at 4°C for 12 hr and embedded in paraffin. Sections (5 μm) were cut and stained with H&E. Images of entire kidney cross-sections from five different renal planes were captured and analyzed using AxioVision (release 4.3) image analysis software (Carl Zeiss Microscopy). All values were normalized to renal area.

*Immunohistochemistry.* Slides were exposed under pressure to Antigen Unmasking Solution® (Vector Labratories, Burlingame, CA). Sections were incubated with Wilms tumor suppressor (WT1) antibody (180 amino acids in length; Santa Cruz Biotechnology, Santa Cruz, CA) or AHR rabbit polyclonal antibodies (Biomol International, Plymouth Meeting, PA) overnight at 4°C in a solution of 0.3% Triton-X and 5–10% goat serum. Primary antibodies were bound to a goat anti-rabbit biotinylated secondary antibody (Invitrogen-Molecular Probes, Carlsbad, CA), amplified with the the Vectastain Elite ABC Kit; Vector Laboratories), developed with diaminobenzidine (DAB), and counterstained with Mayer’s hematoxylin (Vector Laboratories). Threshold optimization was completed relative to negative controls, and indices of protein expression were expressed as sum density normalized to total area. Podocyte numbers were quantified using WT1 signal filtered for color, intensity, and size. All values were normalized to glomerular density.

*Quantitative polymerase chain reaction (PCR)*. Total RNA was extracted using TRIzol® (Invitrogen, Carlsbad, CA) and cDNA synthesized using Super Script II (Invitrogen) per manufacturer’s instructions. Quantitative PCR was performed to detect differences in the ratio of *Wt1* splice variants (±KTS) in response to AHR ligand treatment (Falahatpisheh and Ramos 2003). All primers were designed using Beacon Designer (version 5.1; PREMIER Biosoft, Palo Alto, CA) to create amplicons from 150 to 300 base pairs with an average melting temperature of 55°C. All primers used are listed in Table 1.

**Table 1:** PCR primers used in all quantitative PCR assays.

Gene	Forward primer 5´-3´	Reverse primer 5´-3´
18S		CGTCTGCCCTATCAACTTTCG		GCCTGCTGCCTTCCTTGG
Cytochrome P450 1A1 (*Cyp1a1*)		TCGTGTCAGTAGCCAATGTC		GCATCCAGGGAAGAGTTAGG
Taurine transporter (TauT)		CATCCATCGTCATTGTGTC		AAGTTGGCAGTGCTAAGG
Syndecan-1 (*Sdc1*)		GAGAACAAGACTTCACCTTTG		AGCACTTCCTTCCTGTCC
Paired box gene 2 (*Pax2*)		AGGTTTACATCTGGTCTGG		TAGGAAGGACGCTCAAAG
Epidermal growth factor receptor (*Egfr*)		GAGGAGGAGAGGAGAACTG		GGTGGGCAGGTGTCTTTG
Retinoic acid receptor, alpha (*Rar*α)		CCCAGAAGACTAAAGTTGAC		TGGCAGGTAGTTGTGATG
Secreted frizzled-related sequence protein 1 (*Sfrp1*)		GCAGTTCTTCGGCTTCTA		ATGGAGGACACACGGTTG
Insulin-like growth factor 1 receptor (*Igf1r*)		GTCCCTCAGGCTTCATCC		GAGCAGAAGTCACCGAATC
Insulin-like growth factor 2 receptor (*Igr2r*)		AGTATGTGAACGGCTCTG		TCTGTGATTGTCTGGATAGG
Wingless-related MMTV-integration site 4 (*Wnt4*)		GTAGCCTTCTCACAGTCCTTTG		GGTACAGCACGCCAGCAC
Lim homeobox protein 1 (*Lhx1*)		ACCTAAGCAACAACTACAATC		AACACGGGAGTAGAAAGC
E-cadherin		CGACCCTGCCTCTGAATCC		CTTTGTTTCTTTGTCCCTGTTGG


*Urinary albumin.* Individual urine samples were stored at –80°C in stabilizing buffer (Biotrin International, Dublin, Ireland). One microliter of sample was loaded onto 4–12% NuPAGE Bis-Tris gel (Invitrogen) under reducing conditions and processed for silver staining per the manufacturer’s specifications. Sum density values were calibrated to mouse serum albumin standards ranging from 10 to 0.001 μg/μL.

*Urinary renal papillary antigen 1 (RPA1) and glutathione* S*-transferase Yb1 (GSTYb1) measurements.* Ninety-six–well microtiter plates were conjugated with anti-RPA1 or GSTYb1 IgG. Urine was diluted 1:25 and equilibrated for 1 hr at room temperature before addition of antibody–enzyme conjugate. After substrate development, absorbance was read at 450 nm using 630 nm as a reference. Absorbance was normalized to internal controls and expressed as relative units.

*Western blot analysis.* Protein was extracted using T-PER reagent (Pierce, Rockford, IL) per the manufacturer’s specifications. Samples were run on 4–12% NuPAGE Bis-Tris gels under reducing conditions, transferred to a polyvinyl difluoride membrane, and probed with WT1(180) rabbit polyclonal antibody (Santa Cruz Biotechnology) and horseradish peroxidase–conjugated secondary antibody.

*Statistical analysis.* Statistical significance was determined as noted using Student’s *t*-test, analysis of variance (ANOVA), Wilcoxon rank sums, least significant difference (LSD), and Tukey post hoc tests at the *p* < 0.05 level.

## Results

**Figure 1:**
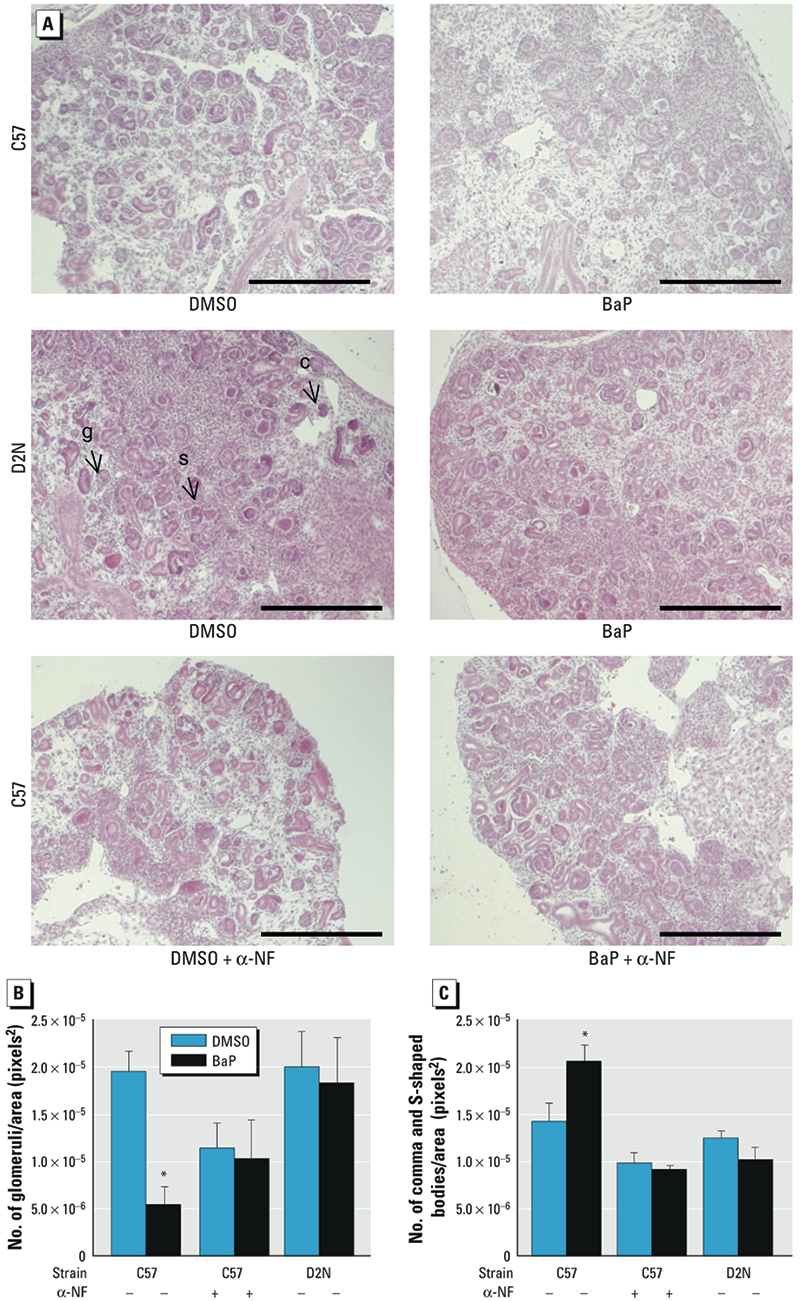
BaP inhibits nephrogenesis via an *Ahr* allele–specific mechanism, as shown by metanephric cultures treated with 3 µM BaP or DMSO without or with α-NF (see “Materials and Methods” for details). (*A*) Photomicrographs of C57 and D2N metanephri stained with H&E. BaP-exposed C57 metanephri display less morphologically distinct differentiated structures compared with the C57 DMSO control. Expression of the *Ahr*^d^ allele in D2N mice abrogates BaP-induced deficits; co‑treatment with the competitive inhibitor α-NF also inhibited BaP effects. Abbreviations: C, comma-shaped bodies; g, glomeruli; s, S-shaped bodies. Bars = 100 µm. (*B* and *C*) Quantification (mean ± SD) of glomeruli (*B*) and comma and S‑shaped bodies (*C*) normalized to area from serial sections (*n* ≥ 6 metanephri/group). *p < 0.05 compared with the corresponding DMSO control, by ANOVA and LSD post hoc tests.

*Deficits in metanephric differentiation by BaP are linked to* Ahr^b-1^. Metanephric explants of C57-*Ahr^b-1/b-1^* and D2N-*Ahr^d/d^* mice were challenged with 3 μM BaP for 1, 3, or 4 days. Quantitative reverse-transcriptase PCR qRT-PCR analysis of C57 metanephri revealed time-dependent induction of cytochrome P450 1A1 (*Cyp1a1*) mRNA, whereas D2N metanephri showed a markedly abrogated response (Table 2). A consecutive 4-day exposure of C57 cultures to BaP significantly delayed metanephric development (Figure 1A), as evidenced by decreased numbers of mature glomeruli (Figure 1B) and corresponding increases in comma and S-shaped bodies, structures indicative of immature glomerular development (Figure 1C).

**Table 2:** *Cyp1a1* mRNA expression.

Exposure (days)	Normalized fold change relative to DMSO control
C57		
1		154.34 ± 20.8
2		455.09 ± 30.62
3		730.00 ± 24.00
D2N		
1		1.82 ± 0.27
2		12.44 ± 2.03
3		39.49 ± 1.20


**Figure 2:**
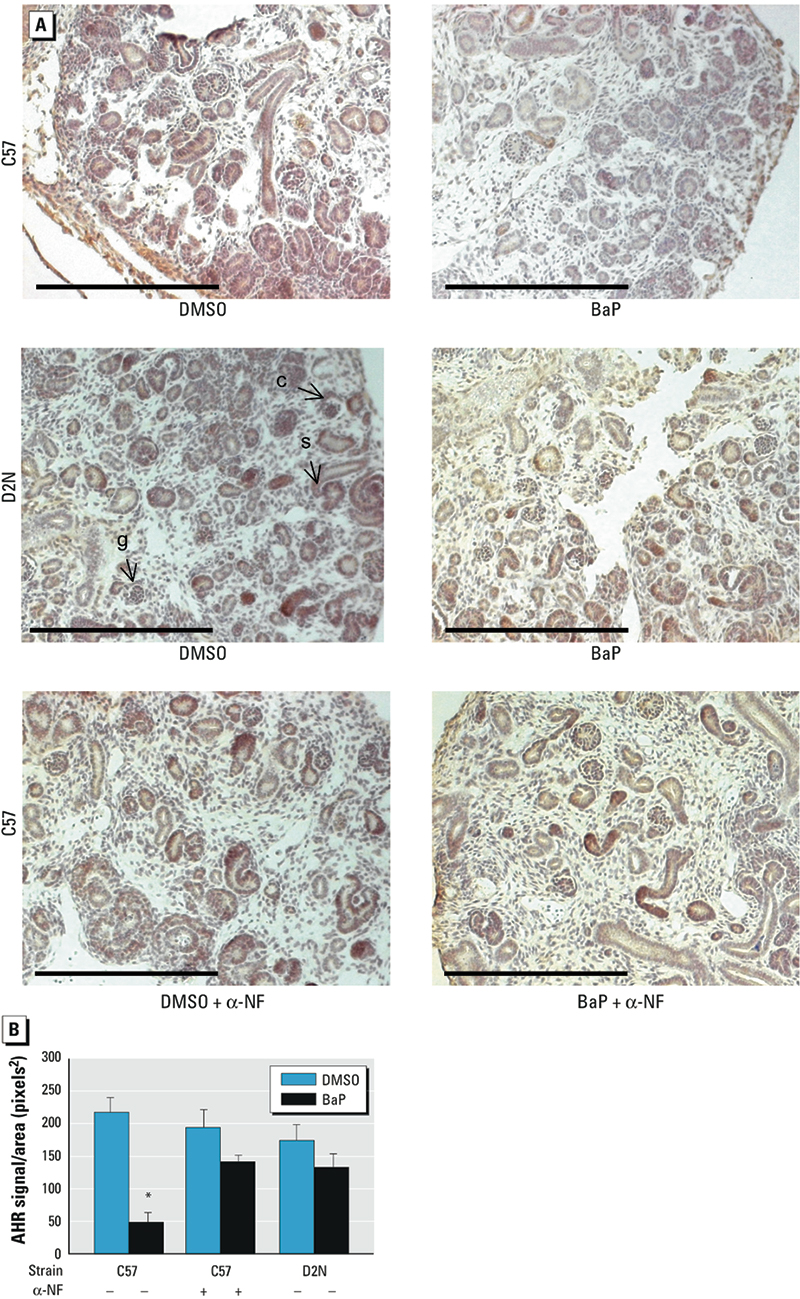
AHR expression correlates with nephrogenesis, as shown by immunohistochemical analysis of metanephric cultures treated with 3 µM BaP or DMSO without or with α-NF (see “Materials and Methods” for details). Exposure to BaP decreases AHR protein levels, as indicated by DAB staining (*A*) and density (mean ± SD) of AHR normalized to total area (*B*). Bars = 100 µm. Protein expression was similar in DMSO-treated C57 and D2N metanephri, but D2N and α-NF–co-treated C57 metanephri were not sensitive to BaP-induced deficits in AHR protein expression. *p < 0.05 compared with the corresponding DMSO control, by ANOVA and LSD post hoc tests.

To determine whether ligand binding to AHR-mediated deficits in renal cell differentiation, we examined effects of BaP exposure on D2N mice expressing the *Ahr^d/d^* allele and C57 mice co-treated with α-NF, a competitive inhibitor at the AHR ligand-binding site. Activation of the low-affinity Ahr^d/d^ receptor was not associated with morphologic or genetic deficits in developing kidneys. Although α-NF slightly decreased glomerular density, we observed no reciprocal changes in the abundance of undifferentiated structures or alterations in gene expression (Figure 1B). As expected, α-NF efficiently neutralized the actions of BaP on metanephric differentiation, as evidenced by reversal of dedifferentiation deficits. Immunohistochemical analysis showed a decrease in AHR protein after 4 days of BaP exposure in *Ahr^b-1/b-1^* mice (Figure 2), whereas the abundance of AHR^d/d^ protein was not influenced by BaP treatment (Figure 2A). Instead, expression of *Ahr^d/d^*, or co-treatment with BaP and α-NF, prevented loss of AHR protein. These findings are consistent with the hypothesis that AHR is required for renal developmental signaling and that disruption of nephrogenesis by BaP requires AHR ligand binding, signaling, and protein degradation.

**Figure 3:**
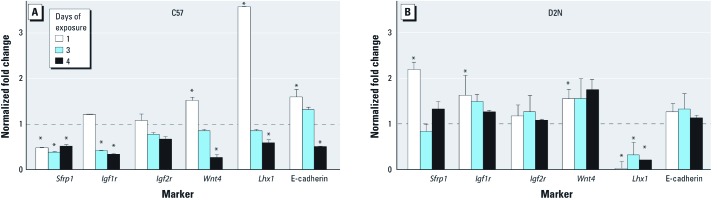
BaP exposure down-regulates markers of renal cell differentiation, as shown by qRT‑PCR analysis of BaP-treated C57 (*A*) and D2N (*B*) metanephric cultures (see “Materials and Methods” for details). Values (mean ± SD) represent 2^−∆∆CT^ normalized fold change relative to the DMSO CF7 control (dashed line); *n* ≥ 6 metanephri/group. At 4 days of BaP exposure, C57 metanephri (*A*) but not in D2N metanephri (*B*) showed significant decreases in differentiation markers. *p < 0.05, by Wilcoxon rank sum test.

Next, we monitored the expression of mesenchymal [*Sfrp1* (secreted frizzled-related sequence protein 1)]and epithelial [*Igf1r* (insulin-like growth factor 1 receptor), *Wnt4* (wingless-related MMTV-integration site 4), *Lhx1* (Lim homeobox protein 1), and E-cadherin] markers of differentiation by qRT-PCR to determine if morphologic deficits correlated with modulation of genetic targets. Markers of renal epithelial cell differentiation (*Igf1r*, *Wnt4*, *Lhx1*, and E-cadherin) were modulated to variable degrees relative to vehicle control after BaP exposure of C57 metanephri for 1, 3, or 4 days (Figure 3A). *Igf1r*, *Wnt4*, *Lhx1*, and E-cadherin were also significantly down-regulated by day 4 of BaP treatment in C57 cultures (Figure 3A), whereas *Sfrp1* was down-regulated at all time points (Figure 3A). Metanephri expressing the *Ahr^d^* allele showed variable degrees of induction in *Sfrp1*, *Igf1r*, and *Wnt4* throughout the exposure period and were completely spared deficits in differentiation, except for changes in *Lhx1*, which showed decreased levels at all time points (Figure 3B). Collectively, these data indicate that nephrogenic deficits induced by BaP require integrity of AHR signaling and involve interference with coordinated renal cell differentiation programming.

**Figure 4:**
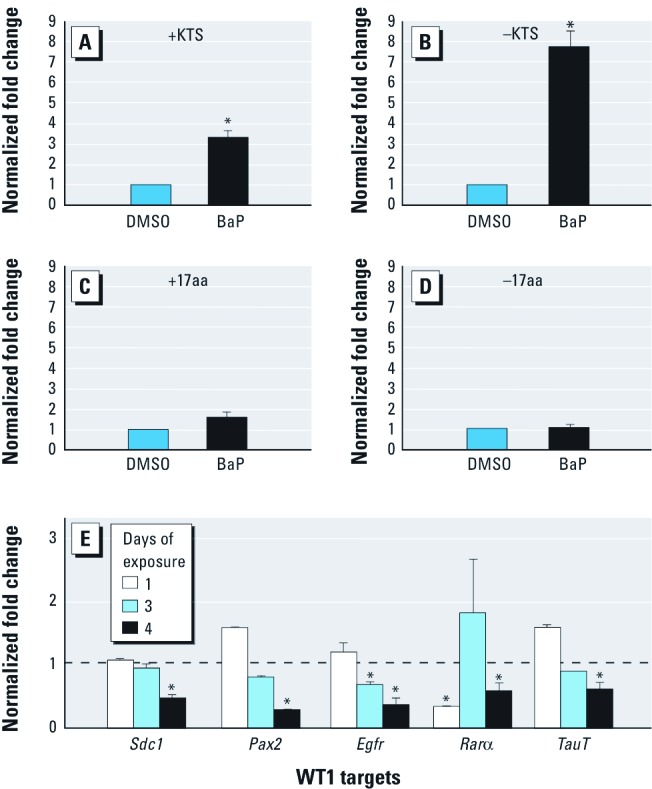
WT1 dysregulation by BaP correlates with loss of known WT1 targets in C57 metanephri, as shown by qRT‑PCR analysis (see “Materials and Methods” for details). Values (mean ± SD) represent 2^−∆∆CT^ normalized fold change relative to the DMSO control (shown as a dashed line in *E*); *n* ≥ 6 metanephri/group. (*A*–*D*) Abundance of splice variants from BaP-treated and DMSO control samples. After 4 days of BaP exposure, significant increases were seen in –KTS (*B*) compared with +KTS (*A*) isoforms but not in +17aa (*C*) or –17aa (*D*) isoforms. No changes were seen in total *Wt1* mRNA expression. (*E*) Down-regulation of known WT1 targets correlated with changes in WT1 isoform abundance. *p < 0.05, by Wilcoxon rank sum test.

**Figure 5:**
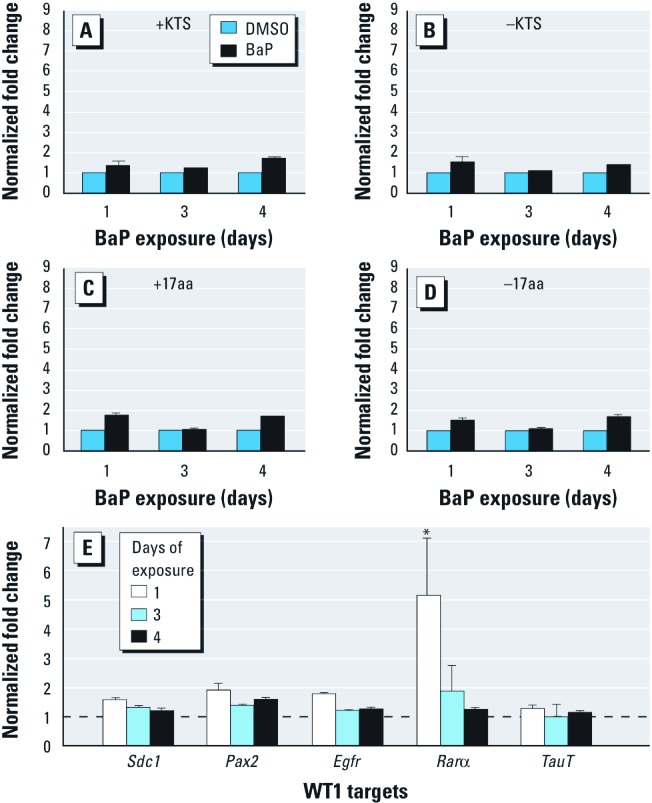
WT1 dysregulation by BaP is *Ahr* allele specific in D2N-Ahr^d/d^ metanephri, as shown by qRT‑PCR analysis (see “Materials and Methods” for details). Values (mean ± SD) represent 2^−∆∆CT^ normalized fold change relative to the DMSO D2N control (shown as a dashed line in *E*); *n* ≥ 6 metanephri/group. (*A*–*D*) Abundance of splice variants [(*A*) +KTS, (*B)* –KTS, (*C*) +17aa, and (*D*) –17aa)] from BaP-treated and DMSO CF7 control samples. Expression of the *Ahr^d/d^* allele abrogated BaP-induced modulation of *Wt1* mRNA isoforms in D2N mice. No changes were seen in total *Wt1* mRNA expression. (*E*) Down-regulation of known WT1 targets correlated with changes in WT1 isoform abundance. 18S, β‑actin, and GADPH were run as internal controls for all qRT-PCR reactions. While all controls tested demonstrated tolerable variability, 18S provided the optimal reproducibility in our assays. *p < 0.05, by Wilcoxon rank sum test.

Ahr *allele mediates disruption of* Wt1 *mRNA splice variants by BaP.* BaP exposure of C57 metanephri for 4 consecutive days resulted in 3- and 8-fold induction of +KTS and –KTS variants, respectively (Figure 4A,B). Expression of +17aa or –17aa was not altered by BaP (Figure 4C,D). Consistent with the known transcriptional repressive activity of WT1, significant reductions in the relative expression of several WT1 targets, including *Sdc1*, paired box gene 2 (*Pax2*), *Egfr*, and *Rar*α, were observed by day 4 of BaP treatment (Figure 4E). D2N-Ahr^d/d^ metanephri exposed to 3 μM BaP did not exhibit changes in any of the *Wt1* mRNA splice variants (Figure 5A–D) or *Wt1* target genes (Figure 5E), except for Rarα, where the pattern of regulation was reversed compared with the C57 strain. C57 but not D2N metanephri exhibited decreased taurine transporter (*TauT*) mRNA (compare Figures 4E and 5E).

**Figure 6:**
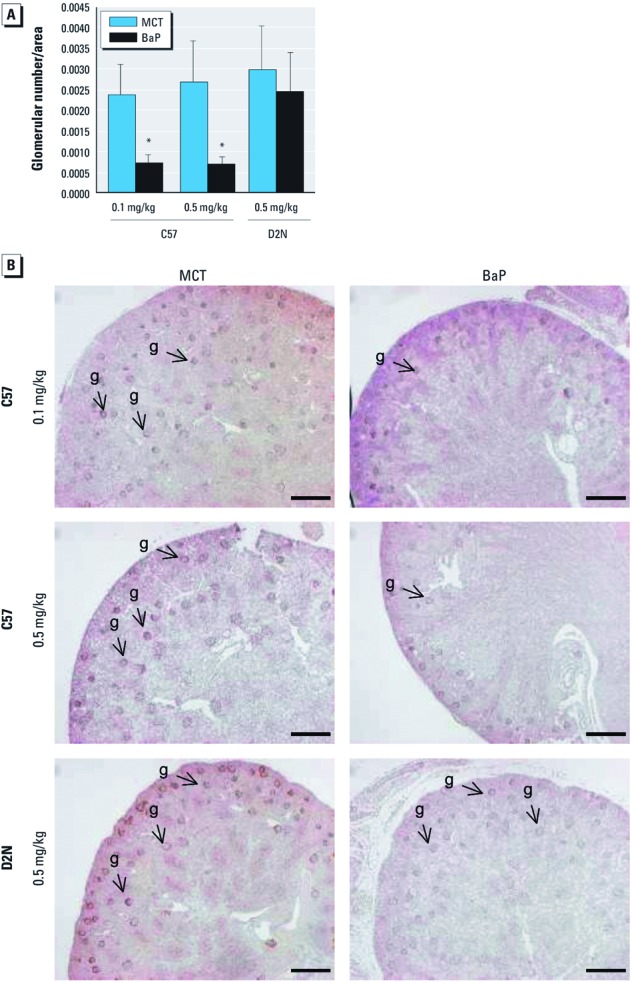
BaP inhibits nephrogenesis via an *Ahr* allele–specific mechanism, as shown by glomerular number normalized per area (mean ± SD; *A*) and photomicrographs of eosin-stained cross sections (bars = 200 μm; *B*) from kidneys resected from 7‑day old C57^b1/b1^ and D2N-AHR^d/d^ mice exposed to 0.1 or 0.5 mg/kg BaP or MCT oil vehicle *in utero *(see “Materials and Methods” for details). g, glomeruli. Bars = 200 μm. *p < 0.05 compared with MCT, by ANOVA and LSD post hoc tests.

*Intrauterine exposures to BaP alter renal development and function of the offspring.* Control C57 and D2N mice showed similar numbers of glomeruli (Figure 6A). *In utero* exposure to both 0.1 and 0.5 mg/kg BaP during GDs 10–13 caused significant reductions in glomerular numbers (Figure 6). One week after birth, the kidneys of offspring of C57 dams exposed to BaP exhibited significant reductions in glomerular size and increased numbers of undifferentiated cells compared with controls (Figure 6B). In contrast, D2N mice expressing the low-affinity *Ahr* allele were spared BaP-induced glomerular deficits (Figure 6).

**Figure 7:**
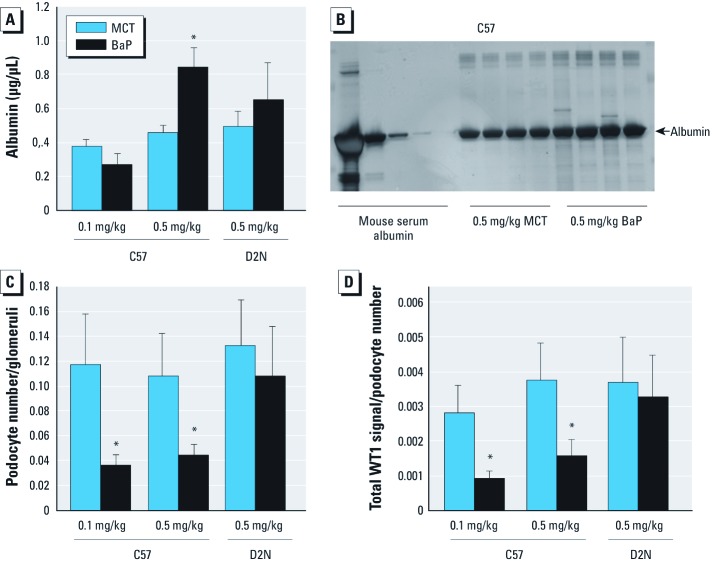
BaP exposure induces glomerular-specific injury, as shown by albumin (*A,B*), podocyte number (*C*), and total WT1 signal (*D*) in urine collected from 52‑week-old C57^b1/b1^ and D2N-AHR^d/d^ mice exposed to 0.1 or 0.5 mg/kg BaP or MCT oil *in utero* (see “Materials and Methods” for details). (*A*) Expression of the *Ahr^d/d^* allele abrogates BaP-induced alterations in albumin urinary levels in C57 mice compared with D2N mice. (*B*) Silver stain visualization of mouse urinary albumin in C57 mice exposed *in utero* to 0.5 mg/kg MCT or BaP. (*C*) Immunohistochemical analysis of podocyte numbers quantified using WT1 signal filtered for intensity, color, and size normalized to glomerular density. (*D*) Immunohistochemical analysis of total WT1 signal quantified using WT1 normalized to podocyte numbers. For *A*, *C*, and *D*, data are mean ± SD. *p < 0.05 compared with the corresponding MCT control, by ANOVA and LSD post hoc tests.

**Figure 8:**
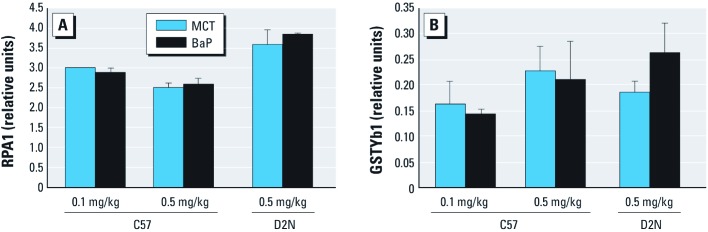
BaP exposure does not induce collecting duct or distal tubular injury, as shown by absorbances from enzyme immunoassay detection of RPA1 (*A*) and GSTYb1 (*B*) in urine collected from 52‑week-old C57^b1/b1^ and D2N-AHR^d/d^ mice exposed to 0.1 or 0.5 mg/kg BaP or MCT oil *in utero* (see “Materials and Methods” for details). Absorbances were normalized to internal controls and expressed as relative units (mean ± SD). *p < 0.05 compared with the corresponding MCT control, by ANOVA and LSD post hoc tests.

C57 and D2N mice exhibited similar amounts of urinary albumin, demonstrating that expression of the *Ahr^d^* allele in itself does not compromise renal function in unstressed animals (Figure 7A). Measurements of urinary albumin 52 weeks after intrauterine exposure to BaP showed dose-dependent increases in urinary albumin only in C57 mice expressing the responsive *Ahr^b-1^* allele, whereas D2N mice were unaffected (Figure 7A,B). Immunohistochemical quantification of podocyte numbers revealed no differences between C57 and D2N mice (Figure 7C). *In utero* exposure to 0.1 or 0.5 mg/kg BaP was associated with decreased podocyte numbers in 52-week-old C57 but not D2N mice (Figure 7C). Consistent with this finding, both doses of BaP decreased WT1 protein expression in an *Ahr* allele–specific manner (Figure 7D). BaP exposure did not alter urinary RPA1 or GSTYb1 levels in either C57 or D2N mice (Figure 8), suggesting that *in utero* BaP exposure was selectively associated with glomerular pathology and did not involve collecting duct (RPA1) or distal tubular injury (GSTYb1) (Falkenberg et al. 1996; Hildebrand et al. 1999; Kilty et al. 1998).

## Discussion

Significant progress has been made in understanding the genetic basis of renal developmental disease. Of note is the discovery that mice overexpressing the –KTS isoform of *Wt1* have severely compromised renal development, increased stromal tissue, decreased tubular epithelium and glomeruli, and altered podocyte structure (Hammes et al. 2001). Although the exact molecular consequences resulting from alterations of the four predominant and biologically relevant isoforms of WT1 remain to be defined, it is well established that the –KTS mRNA encodes a protein that participates in transcriptional regulation, whereas the protein encoded by +KTS mRNA associates with splicing factors (Hastie 2001). Here we present evidence that BaP induces shifts in –KTS that correlate with down-regulation of differentiation markers. The actions of BaP are likely mediated by unregulated activation of AHR signaling during nephrogenesis and involve the classical AHR–HIF1β macromolecular complex or molecular interactions with NF-κB (nuclear factor κB), activator protein 1 (AP-1), and/or glucocorticoid receptor (Nanez and Ramos 2010). Of relevance are reports showing that the glucocorticoid receptor associates with peroxisome proliferator-activated receptor gamma coactivator-1 (PGC-1), a known regulator of cotranscriptional splicing (Knutti et al. 2001). Another possibility involves direct binding of AHR to the *Wt1* promoter, which contains two consensus AHR-responsive elements within a regulatory region known to regulate transcription-coupled splicing events (Cohen et al. 1997). Because AHR associates with proteasomal complexes consisting of damaged-DNA-binding protein 1, aryl hydrocarbon receptor nuclear translocator, transducin-β-like 3, and cullin 4B and itself possesses E3 ligase activity (Ohtake et al. 2007), AHR may regulate proteasomal degradation of factors required for *Wt1* splicing. Although the E3 ligase activity of AHR has not been fully characterized, AHR ubiquitination occurs in both cytoplasm (Song and Pollenz 2002) and nucleus (Marlowe and Puga 2010).

A model detailing a constitutive function for AHR in the regulation of nephrogenesis via WT1 is further supported by our finding that use of the genetically resistant D2N strain, which expresses the *Ahr^d^* allele or competitive antagonists of AHR, prevented renal developmental deficits in BaP-treated metanephri and restored normal ratios of *Wt1* mRNA isoforms. The modulation of renal differentiation markers in organ culture induced by BaP is consistent with the deficits in kidney morphogenesis observed after intrauterine exposure to BaP and with mounting evidence implicating AHR in the regulation of proliferation, development, adhesion, migration, and proteasomal degradation of several organ systems (Nanez and Ramos 2010). Mutation of the ligand-binding domain in the *Ahr^d^* allele decreases ligand-binding affinity by 10-fold compared with *Ahr^b-1^* and decreases nuclear translocation efficiency, transcriptional activation, and degradation upon ligand binding (Poland et al. 1994). Likewise, α-NF does not elicit the conformational changes necessary for efficient translocation and/or protein degradation (Henry and Gasiewicz 2003). *Ahr*-null mice exhibit delayed nephrogenesis and compromised renal development (Falahatpisheh and Ramos 2003) and a hypertensive phenotype (Lund et al. 2003, 2006), so the renal phenotype of *Ahr*-null mice, as well as that of mice treated *in utero* with BaP, may be mediated by interference with developmental programming at the metanephric stage of renal cell differentiation. Together, our findings implicate AHR as a key regulator of renal morphogenesis and differentiation and suggest that exogenous ligands of AHR alter genetic programming of the kidney during critical periods of development.

The disruption of nephrogenesis by BaP involved changes in expression of epithelial markers of differentiation. Sfrp1 is a mesenchymal marker expressed in the metanephric medullary and cortical stroma that acts as a regulator of branching morphogenesis and tubule formation (Yoshino et al. 2001). Igf2r is expressed during early nephrogenesis, whereas Igf1r is expressed ubiquitously throughout renal maturation (Duong Van Huyen et al. 2003), and loss of either receptor compromises renal growth and differentiation (Feld and Hirschberg 1996). Wnt4 is expressed in condensed mesenchyme and pretubular aggregates (Vainio et al. 1999), and Wnt4 deficiency inhibits tubule development (Stark et al. 1994). Lhx1 is chiefly expressed in the ureteric bud and induced in mesenchymal aggregates and differentiating comma- and S-shaped bodies (Barnes et al. 1994; Karavanov et al. 1998), and murine models of Lhx1 deficiency are born headless and without kidneys despite the presence of other organs (Shawlot and Behringer 1995). *Lhx1*-null mice do not progress past mesonephric development (Shawlot and Behringer 1995). E-cadherin is a general marker of mesynchemal-to-epithelial cell transition that is expressed in the ureteric bud epithelium, distal tubule progenitor cells, and most differentiated tubular epithelium (Cho et al. 1998). E-cadherin–null mice show decreased nephrons due to a failure of proper fusion of metanephric mesenchyme to the ureteric bud (Mah et al. 2000). The critical functions executed by these genes during metanephric differentiation and the disruption of coordinated patterns of gene expression induced by BaP in an AHR-specific manner further implicate the AHR as a critical regulator of renal cell differentiation.

Fetal programming is a process whereby during a critical window of development a stimulus induces lasting effects on the structure or function of the organism (Barker 2004). The evidence we present here shows that intrauterine exposure to BaP during nephrogenesis is associated with sustained deficits of renal structure and function that compromise organ function long after birth. These observations are consistent with previous correlations linking low birth weight (LBW) and smoking with the disruption of renal developmental programming. LBW humans have reduced nephron numbers and glomerular hypertrophy and are prone to microalbuminuria, proteinuria, and decreased glomerular filtration (Celsi et al. 1998; Nwagwu et al. 2000; Sanders et al. 2005). Reduced renal capacity leads to glomerular hypertension and compensatory hypertrophy, which in turn is associated with disruption of the glomerular basement membrane and glomerulosclerosis. Sustained glomerular injury exacerbates nephron loss, further reducing renal glomerular filtration rate. A reduction in renal capacity further increases blood pressure and completes a futile cycle of glomerulosclerosis and nephron loss that may ultimately result in progressive renal failure (Zandi-Nejad et al. 2006).

Our model in which maternal BaP insult results in decreases in podocyte numbers that impair glomerular filtration is strikingly similar to clinical manifestations seen in conditions such as focal segmental glomerulosclerosis or diabetic nephropathy (Hara et al. 2001; Hayden et al. 2005; Kim et al. 2001; Wolf et al. 2005). Podocytes are terminally differentiated cells that line the glomerular basement membrane and act as a size-selective filter that must structurally encompass the entire glomerular surface with foot processes to maintain proper filtration (Wiggins 2007). Podocytopenia results in denudation of the basement membrane that compromises glomerular function to initiate a cycle of injury and continued podocyte loss, leading to progressive renal failure. In fact, glomerulopathies are the most common causes of end-stage renal disease worldwide (Hricik et al. 1998).

## Conclusions

The discovery of novel functions of AHR during nephrogenesis highlights a potential mechanism for compromised renal development after disruption of AHR signaling in pregnant women exposed to tobacco smoke or babies exposed postnatally to second-hand smoke. Such bioavailability of polycyclic aromatic hydrocarbons provides a ready source for agents that modify early determinants of renal cell differentiation. Of relevance are studies linking *AHR* genetic polymorphisms to tobacco-smoke–induced decreases in birth weight (Sasaki et al. 2006), along with associated microalbuminuria, proteinuria, and heightened risk of end-stage renal disease (Zandi-Nejad et al. 2006). Other stressors, such as diet and environmental exposures, are associated with LBW and deficits in renal development (Everson et al. 1988; Nelson et al. 1999; Roquer et al. 1995; Zandi-Nejad et al. 2006). In humans, strong associations have been reported between smoking and albuminuria in nondiabetic patients (Pinto-Sietsma et al. 2000) and a higher risk of renal dysfunction and renal cancer (Bertazzi et al. 1989). The protection afforded in mice by *Ahr^d^* suggests that the risk of AHR-mediated renal developmental deficits is most likely relevant in subjects expressing polymorphic variants of *AHR* with increased ligand affinity. Such deficits are not severe but instead may compromise renal reserve capacity, leading to increased susceptibility to late-onset renal disease.
